# Prophylactic feeding of neomycin to Holstein calves alters gut microbiota, bile acid metabolism, and expression of genes involved in immunometabolic regulation

**DOI:** 10.3389/fmicb.2023.1210142

**Published:** 2023-08-31

**Authors:** Lautaro R. Cangiano, Ignacio R. Ipharraguerre, Le Luo Guan, Lauralise N. Buss, Rocio Amorin-Hegedus, Miguel Chirivi, G. Andres Contreras, Michael A. Steele

**Affiliations:** ^1^Department of Animal Biosciences, University of Guelph, Guelph, ON, Canada; ^2^Institute of Human Nutrition and Food Science, University of Kiel, Kiel, Germany; ^3^Department of Agricultural, Food and Nutritional Science, University of Alberta, Edmonton, AB, Canada; ^4^Genetics Institute, University of Florida, Gainesville, FL, United States; ^5^Department of Large Animal Clinical Sciences, Michigan State University, East Lansing, MI, United States

**Keywords:** antibiotics, dysbiosis, dyslipidemia, bile acids, metabolism

## Abstract

The objective of this study was to evaluate the effects of prophylactic neomycin administration on Holstein bull calves’ intestinal microbiota, bile acid (BA) metabolism, and transcript abundance of genes related to BA metabolism. A total of 36 calves were blocked by body weight and assigned to either non-medicated milk replacer (CTL), or neomycin for 14 days (ST) or 28 days (LT) in their milk replacer. At the end of the study, calves were euthanized to collect tissue and digesta samples from the gastrointestinal tract, liver, and adipose tissue for analysis of intestinal microbial diversity, bile acid concentration and profile in various body tissues, and gene expression related to bile acid, lipid, carbohydrate metabolism, and inflammation. Calves that received prophylactic administration of neomycin for 28 d (LT) had reduced species richness (chao1 index), and tended to have reduced phylogenetic diversity in the ileum tissue. The relative abundance of *Lactobacillus*, and *Bifidobacterium* in ileum and colon digesta were decreased in LT compared with CTL. Concentrations of primary, secondary, and total BA were increased by ST in ileal tissue. In plasma, ST and LT treatments had lower concentrations of secondary BA. Gene expression of the BA receptor *FXR* was increased in ileum and liver by LT compared to CTL. The expression of *FXR* and *TGR5* in the liver was increased in the ST group compared with CTL, and in adipose tissue, 5 genes related to triglyceride, gluconeogenesis, and immune activation were differentially expressed between CTL and ST. In conclusion, we provide evidence that prophylactic administration of neomycin leads to aberrant changes in BA concentration and profile in different compartments of the enterohepatic system through a process that possibly entails antimicrobial disruption of key bacterial groups, which persists even after cessation of neomycin administration. Additionally, we uncovered an apparent link between dysregulated BA metabolism and changes in lipid metabolism and immune activation in adipose tissue and liver.

## 1. Introduction

The preweaning and weaning periods remain amongst the most challenging stages of a calf’s life, as reflected by high morbidity and mortality rates. It is estimated that 5–6% of female dairy calves die during the preweaning period on farms across Canada and the United States (Urie et al., 2018; Winder et al., 2018). Moreover, male dairy calves are in an even more trying situation with mortality rates ranging from 5 to 10% on veal farms (Pempek et al., 2018). Diarrhea remains the most common cause of morbidity and mortality for dairy calves, accounting for over half of reported illnesses and one-third of deaths (Urie et al., 2018; Winder et al., 2018). Traditionally, antimicrobials have been used to prevent and treat diarrhea during this period, with 75% of calves being treated with antimicrobials in the United States (Urie et al., 2018) and 30% in Canada (Uyama et al., 2022). The preventative use of prophylactic antimicrobials is a common practice in calf rearing. However, the demonstrated low efficacy of this practice to prevent disease, in addition to concerns regarding antimicrobial resistance (Windeyer et al., 2014), have called for its revision in recent years. In a companion study conducted with the same cohort of animals as in this study, we demonstrated that although prophylactic dosing of neomycin in milk during preweaning resulted in a small reduction in fecal scores, it did not reduce incidence of diarrhea or respiratory disease and did not result in improved animal performance (Buss et al., 2021).

After birth, the animals undergo a series of developmental adaptations to the new environment and the acquired intestinal bacteria help stimulate intestinal and immune development (Hooper et al., 2012). Alterations caused by environmental factors, such as antimicrobial use, can have lasting implications on health and productivity (Heinrichs and Heinrichs, 2011; Aghakeshmiri et al., 2017). Although no studies have directly investigated the impacts of antimicrobial use during the preweaning period on development, epidemiological data suggests that antimicrobial treatment during the preweaning period is correlated with lower performance and increased incidence of disease later in life. Calves treated with antimicrobials during this period of life have lower conception rates, increased time to first calving, increased risk of leaving the herd earlier due to health complications, and reduced milk production during their first lactation. This suggests that antimicrobial administration during early life might impact animal development and health in ways not previously anticipated (Svensson and Hultgren, 2008; Abuelo et al., 2021).

Antimicrobial use during the neonatal phase can result in changes in functional and structural diversity of the intestinal microbiota. In mice, reduced microbial diversity in early life can lead to dyslipidemia and increased fat deposition (Bäckhed et al., 2004), and in farm animals, antimicrobials are routinely used to improve efficiency of weight gain. One of the metabolic axes that seem to be disrupted by antibiotic use is the metabolism of bile acids (BA; Ipharraguerre et al., 2018). Besides their classical role as lipid emulsifiers, BAs act as pleiotropic signaling molecules, playing a role in nutrient sensing during the postprandial state by alerting peripheral tissues of incoming nutrients, ultimately modulating glucose homeostasis and energy expenditure (De Aguiar Vallim et al., 2013; Kuipers et al., 2014). Bile acids are involved in the systemic regulation of several metabolic and inflammatory pathways, and their function is affected by the composition of the intestinal microbiota (Cai et al., 2022). Secondary bile acids (2BA) produced by bacterial modification of primary BA (synthesized by the liver) lead to changes in their agonistic function on target receptors in the intestine, liver, and peripheral tissues (Chávez-Talavera et al., 2017; Cai et al., 2022). The profile and concentration of these BA largely depend on the composition of the intestinal microbiota, establishing a direct link between the intestinal microbiota and the regulation of metabolic homeostasis. The loss in functional diversity of the intestinal microbiota caused by antimicrobials alters the biosynthesis of BA leading to changes in their “instructive functions” on metabolism and immunity in peripheral tissues (Ipharraguerre et al., 2018). Therapeutic and subtherapeutic antimicrobial use has been shown to alter BA profile, tissue distribution, and expression of downstream genes involved in energy metabolism and immune function in rodents (Swann et al., 2011; Cai et al., 2022) and piglets (Ipharraguerre et al., 2018), ultimately altering lipid metabolism. To date, no studies have looked at the effects of prophylactic administration of antimicrobials on intestinal function and its effects on systemic metabolism. Therefore, the objective of this study was to evaluate the impact of prophylactic administration of the antimicrobial neomycin to Holstein bull calves on the intestinal microbiota, BA metabolism, and expression of genes related to immunity and energy metabolism in peripheral tissues. We hypothesized that prophylactic antimicrobial administration induces changes in the intestinal microbiota leading to aberrant intestinal development and altered signaling within the gut-liver axis, ultimately compromising peripheral tissue metabolism.

## 2. Materials and methods

### 2.1. Animals, housing, and treatments

The experiment was conducted as per the guidelines of the Canadian Council of Animal Care (CCAC, 1993) at a commercial veal facility (Mapleview Agri Ltd., Palmerston, ON, Canada), and approved by Animal Care Committee at the University of Guelph (Animal Use Protocol #4134). The animal protocol and experimental design were already described in a companion study looking at the effects of neomycin on health and performance of Holstein bull calves (Buss et al., 2021). Briefly, a total of 36 calves (6 days old ±3) split across two experimental periods were enrolled in the study from May to September 2019 (*n* = 18 per period). Upon arrival, calves were individually housed in 78 × 120 cm pens for the remainder of the experiment. Calves received two meals of a standardized 26:17 milk replacer (MR) composed of 26% CP, 17% crude fat, 45% lactose, 8% ash, 0.25% fiber, 2.1% Lys, 0.8% Met, and 1.5% Thr 4.66 Mcal/kg of ME, on a DM basis. The MR was fed at 530 g/MR per meal at 13% solids for the first 2 weeks and increased to 780 g/MR per meal at 15% solids on d 14 of the study until the end of the study on d 28. Calves had *ad libitum* access to texturized solid feed and water.

A generalized randomized block design was used to evaluate the impact of prophylactic neomycin administration in milk on BA metabolism and transcript abundance of genes involved in BA sensing, lipid metabolism, and inflammation. Calves were blocked by body weight (BW) and assigned to one of three treatments: control (CTL: non-medicated MR), short-term (ST: neomycin for 14 days fed in MR), or long-term antimicrobial (LT: neomycin for 28 days fed in MR). Neomycin was delivered in a soluble powder form (81.25% neomycin sulfate; Neomed 325, Bio Agri Mix, ON, Canada) to the ST and LT groups by mixing into MR at a dose of 20 mg/kg of BW (based on current Food and Drug Administration regulations regarding the use of neomycin in milk replacers; Food and Drug Administration, 2020). Researchers were blinded to treatment groups for the duration of the study. Only barn staff responsible for the administration of neomycin were aware of treatment groups.

Health scoring was performed by a trained individual on a daily basis. Fecal scoring was completed twice daily before milk feedings for the 28 days of the study using the following scoring system: 0 = solid, 1 = soft, 2 = pasty/runny, 3 = runny/watery (Larson et al., 1977). A healthy score was considered 0 or 1, and abnormal considered 2 or 3. Time to first diarrhea was defined as the number of days until a calf had a fecal score of 2 or 3. To comply with standards of animal health and welfare, the use of therapeutic antimicrobials for treatment of disease was unavoidable in this study. The therapeutic use of antimicrobials was based on a health scoring criterion described in Buss et al. (2021) and used as the last resource in the event of confirmed bacterial infection. All treatments were recorded and only injectable antimicrobials were used.

### 2.2. Intestinal tissue and gut digesta sampling

On d 29 ± 1, calves were euthanized *via* captive bolt and exsanguination to allow for collection of tissue and digesta samples from the gastrointestinal tract. The abdominal cavity was opened, and the rectum and esophagus were ligated before removing the gastrointestinal tract to prevent loss of digesta. Segments of the distal jejunum, ileum, and colon, along with their digesta were collected according to Welboren et al. (2021). Briefly, the distal jejunum segment was taken 30 cm proximal to the collateral branch of the cranial mesenteric artery; the ileum segment was taken 30 cm proximal to the ileo-cecal junction; and the colon segment was collected 30 cm distal to the ileo-cecal junction. Zip-ties were placed on each end of the segments so that a sample of digesta could be collected. Intestinal digesta were removed from the sample using tweezers and placed in a 50-mL Falcon tube. The tissue was then washed in sterile PBS (pH 7.4) until clean (~3–4 washes) and placed in a sterile bag. Both gut digesta and tissue samples were immediately snap-frozen in liquid nitrogen and then stored at −80°C until further processing. In addition, an approximately 16 cm^2^ sample was taken from the middle of the right lobe of the liver, 15 cm away from the falciform ligament. Lastly, a subcutaneous sample of peripheral adipose tissue was taken from the rump cap area, close to the base of the tail, and a sample of omental fat from the left of the collateral branch of the cranial mesenteric artery. Both gut digesta and tissue samples were immediately snap-frozen in liquid nitrogen and then stored at −80°C until further processing.

### 2.3. Bile acid sample preparation and determination by LC–MS

In plasma samples (100 μl), proteins were precipitated with 10 μl of deuterated BA internal standards (IS, Steraloids, Newport, R.I.) and diluted with 300 μl of a 1:1 acetonitrile:distilled water solution. After vortexing and incubation at room temperature for 10 min, samples were centrifuged at 15.000 *g* × 10 min, at 4°C. Supernatant was collected and completely evaporated and the solid residue was reconstituted with 100 μl of H_2_O/MeOH 1:1 and directly injected in the liquid chromatographer coupled to a mass spectrometer (LC–MS). Intestinal digesta samples (40 mg) were mixed with 400 μl of a 1:1 acetonitrile:distilled water solution and incubated at room temperature for 15 min. The following steps were performed as described for plasma samples. Intestinal tissue samples (200 mg) were first homogenized by repeated bead beating (Tissue Lyser 2, Qiagen). Then 100 mg of tissue were mixed in a new 2-ml tube with either 700 μl (ileum) or 500 μl (colon) of IS solution in H2O/ACN 1:1, followed by centrifugation at 15.000 for 15 min at 4°C. Supernatant was diluted in H2O/ACN 2:3 and filtered through a 0.2 μm syringe filter. Liver samples (200–250 mg) were homogenized by repeated bead beating (Tissue Lyser 2, Qiagen) and 50 mg of tissue were mixed with 600 μl of IS solution in H2O/ACN 1:1 in a 2-mL tube. Centrifugation and filtration were later performed the same way as for intestinal tissue.

Determination of BAs was performed as described by Li et al. (2013) and Ipharraguerre et al. (2018), with modifications. Analyses were performed in a HPLC (Shimadzu Prominence) connected to a LTQ-Orbitrap Velos mass spectrometer (Thermo Fisher) operating in full scan negative mode. Chromatographic separation was achieved using a reverse-phase column (Phenomenex C18AQ, 2.0 × 150 mm, 4.0 μm, 80 Angstrom) and mobile phases comprising water +0.1% formic acid (A) and acetonitrile +0.1% formic acid (B). Flow rate was set to 0.2 ml/min, oven temperature to 40°C, and chamber temperature to 10°C. Stepwise gradient was programmed as follows: 0 min, 100% A; 3 min, 100% A; 13 min, 40% A + 60% B; 18 min, 40% A + 60% B; 22 min, 100% B; 32 min, 100% B; 32.5 min, 100% A; 37 min, 100% A. The operating conditions of the MS were: ionization mode = ES(−); capillary = 4.5 kV; ion transfer tube temperature = 350°C; HESI needle temperature = 350°C; gas flow = 900 L/h; mass range = 100–1,200 m/z. Chromatographic peak alignment, peak identification, and peak area finding was performed with MAVEN software (Melamud et al., 2010). Bile acids were identified by comparison of their exact mass, retention times, isotopic distribution and MS/MS fragmentation based on standard curves using deuterated primary and secondary BA as IS. Quantification was performed by normalization of the peak area of each detected BA against the internal standard and by the weight of tissue extracted.

### 2.4. RNA extraction and gene expression analysis

The RNA from adipose tissue samples was extracted according to Chirivi et al. (2022). Samples were thawed in ice and transferred into screwcap tubes containing 1 ml of TRI Reagent (R2050–1 ZymoResearch). The samples were placed on dry ice and homogenized 2–3 times at 3.4 m/s for 30 s using a bead mill tissue homogenizer (Fisher Scientific). Samples were then centrifugated at 12,000 × *g* for 10 min at 4°C, and the liquid phase was collected, avoiding the lipid layer on top. Next, 200 μl of chloroform was added, and the samples were shaken for 15 s and placed on ice for 3 min. Samples were centrifugated for 15 s and the aqueous phase was collected and transferred to the Quick-RNA Miniprep plus kit (R1058, Zymo Research) to extract total RNA according to the manufacturer’s protocol. To eliminate genomic DNA, 80 μL of DNase I (E1010 Zymo Research) was used.

For RNA extraction from the distal jejunum, ileum, colon, and liver, frozen tissue was ground in liquid nitrogen using a mortar and pestle. RNA was extracted as described by Welboren et al. (2021) using the PureLink RNA Mini Kit (Invitrogen). Briefly, 1 mL of TRIzol was added to 70–100 mg of ground tissue, after which the sample was homogenized using a vortex. Then, RNA was extracted and precipitated by adding chloroform, followed by 70% ethanol to the tissue homogenate, and collected using a Spin Cartridge (included in the kit). The samples were treated with a DNase mixture (containing DNase I Reaction Buffer, RNase-free water, and DNase I; Thermo Fisher Scientific) to remove any residual DNA. The Spin Cartridge was washed with wash buffers, and RNA was dissolved in RNase-free water. The concentration of RNA from intestinal, liver, and adipose tissue samples were measured using UV–visible spectroscopy (Nanodrop One Microvolume, Thermo Fisher Scientific). Integrity of the RNA was measured by electrophoresis (TapeStation, Agilent Technologies). All samples had a 260:280 nm ratio between 1.89 and 2.02 and an RNA integrity number higher than 6. The RNA was stored at −80°C. Reverse transcription was performed with 500 ng of RNA using 4 μL of the qScript cDNA SuperMix (95,048 Quantabio) for 5 min at 25°C, 30 min at 42°C, and 5 min at 85°C. The cDNA was stored at −20°C.

For adipose tissue samples, transcriptional studies were performed using the Wafergen SmartChip Real-time PCR system, as described in De Koster et al. (2018). Samples were assayed in duplicate on the high-throughput quantitative PCR instrument Wafergen Smartchip (Takara Bio). SYBR gene expression primers were used for quantitative PCR assays commercially available or designed from bovine sequences and synthesized (IDT-Integrated DNA Technologies). Each 100 nl PCR reaction contained 1× of LightCycler 480 SYBR Green Master Mix (Roche), 200 nM of primer assay, and 2 ng/μl of sample cDNA. To validate the absence of genomic DNA and primer-dimer formation that could produce false-positive results, non-template control and non-reverse-transcriptase controls were included. The following cycling conditions were used on the Wafergen SmartChip Real-time PCR system: initial enzyme activation at 95°C for 10 min, 45 cycles of denaturation at 95°C for 10 s, and annealing at 60°C for 53 s.

For RNA from intestinal tissues and liver, qRT-PCR was performed using a StepOnePlus Real-Time PCR System (Applied Biosystems). To each well, 5 μL of cDNA, 10 μL of DNA polymerase-containing supermix (SsoAdvanced Universal Inhibitor-Tolerant SYBR Green, Bio-Rad), 0.8 μl of 5 μM forward and reverse primer mix, and 4.2 μl of nuclease-free H2O was added. The 2-step qPCR program consisted of 3 min at 98°C, followed by 40 cycles of 10 s at 98°C and 30 s at 60°C. Then, a melt curve was generated at 95°C for 15 s, 60°C for 1 min, and 95°C for 15 s to confirm specificity of the PCR amplicon. In both analyses, NormFinder software (Andersen et al., 2004) was used to select the housekeeping genes based on the lowest pairwise variation value. For adipose tissue, we selected EIF3K (eukaryotic translation initiation factor 3 subunit K), RPLP0 (ribosomal protein Lateral Stalk Subunit P0), and RPS9 (ribosomal protein S9); and for intestinal tissue and liver β-Actin (Actin beta), GAPDH (Glyceraldehyde 3-phosphate dehydrogenase), and RPL19 (ribosomal protein L19) were selected. The expression of genes of interest was normalized against the geometric mean of selected housekeeping genes, as described by Hellemans et al. (2007).

### 2.5. DNA extraction, library preparation, and 16S rRNA gene amplicon sequencing

Extraction of microbial genomic DNA from intestinal digesta and tissue was performed similarly to that previously reported by Yu and Morrison (2004) and Villot et al. (2020). Briefly, the digesta sample (~0.3 g ± 0.1 g) was washed twice with Tris-EDTA buffer. After the addition of cell-lysis buffer containing 4% SDS, samples were subjected to physical disruption at 5,000 rpm for 3 min using Biospec Mini Beads Beater 8 (BioSpec, Bartlesville, OK), followed by incubation at 70°C for 15 min and centrifugation for 5 min at 16,000 × *g* at 10°C. The bead beating, incubation, and centrifugation were repeated once and impurities were removed from the supernatant using 10 M ammonium acetate, followed by DNA precipitation using isopropanol. After precipitation, DNA was further purified using QIAmp fast DNA stool mini kit (Qiagen Inc., Germantown, MD). The quantity and purity of DNA were evaluated using Qubit, as per manufacture recommendations (Invitrogen, Thermo Fisher), and DNA was stored at −20°C until further use.

Library preparation and sequencing were conducted at Genome Quebec (McGill University, Montreal, QC, Canada). Briefly, a fragment of the 16S rRNA gene spanning the V3–V4 hypervariable region was amplified by PCR using dual index (forward and reverse primers), with the forward primer 341F (CCTACGGGNGGCWGCAG) and the reverse primer 805R (GACTACHVGGGTATCTAATCC). The quantity of purified PCR products was evaluated using a NanoDrop 1000 (NanoDrop Technologies, Wilmington, DE) to ensure that the concentration of DNA from all samples was higher than 25 ng/μl. A 16S V3–V4 PCR product library was then prepared using the Nextera XT Index (Illumina) and sequenced to generate paired-end 2 × 300 bp on the Illumina MiSeq platform according to the manufacturer’s instructions. The adapter sequences were trimmed from the raw fastq files, and the trimmed reads were demultiplexed according to the samples using the bcl2fastq2 conversion software version 2.20.0 (Illumina).

### 2.6. Bioinformatic and statistical analysis

The adapter sequences were trimmed from the raw fastq files, and the trimmed reads were demultiplexed according to the samples using the bcl2fastq2 conversion software version 2.20.0. (Illumina). For bioinformatic analysis, the sorted reads were imported and processed using the Quantitative Insight into Microbial Ecology (QIIME2) package version 2021.2 (Boylen et al., 2019). First, low-quality reads (Phred score < 20) and short (<150 bp) reads were filtered out. This was followed by denoising and merging using the plugin DADA2 to generate an amplicon sequence variant (ASV) feature table. Chimeric sequences and singleton ASVs were excluded from further analyses. Species diversity, alpha and beta diversity were performed using QIIME2 with a sampling depth of 1,400. Alpha-diversity analyses were conducted with standard diversity metrics accessed *via* QIIME2, including Chao1, Shannon index, and Phylogenetic diversity (PD) index. A nonparametric analysis of variance (Kruskal-Wallis test) adjusted for multiple comparisons was used to test differences in α-diversity among treatment groups and to calculate *p*-values. Beta diversity was calculated based on the weighted UniFrac distances and non-metric multidimensional scaling (NMDS) was applied to the resulting distance matrix. Analysis of similarity (ANOSIM) was used to calculate *p*-values and to test differences in *β*-diversity among the different days for significance. Finally, the non-parametric Kruskal–Wallis test was used to assess treatment effects in the relative abundance of bile-acid metabolizing bacteria in ileum and colon digesta and tissue and adjusted for multiple comparisons. Statistical significance was declared at *p* ≤ 0.05 and a tendency was considered at 0.05 < *p* ≤ 0.10.

For normally distributed, non-microbial data, data were analyzed using a generalized linear mixed-effect model (SAS version 9.4, SAS Institute Inc.). For repeated measures, the model included the fixed effects of treatment, time, and treatment × time interaction, and the random effect of period, and calf nested within treatment. The covariance structure with the lowest Akaike’s information criterion was selected for each variable. For data collected at single time points, the same generalized linear mixed-effect model without repeated measures was used. The Kenward–Roger method was used to calculate the approximate denominator degrees of freedom for the F tests in the statistical models. Continuous data were examined for normal distribution of residuals after fitting the statistical models using Shapiro–Wilk and homogeneity of variance by plotting residuals against predicted values. Statistical significance was considered at *p* ≤ 0.05 and a tendency was considered at 0.05 < *p* ≤ 0.10.

Lastly, to understand if the changes in alpha diversity and bacterial abundance partially accounted for the changes in bile acid profile and concentration in the intestinal tissue and digesta, we performed a non-parametric Spearman rank correlation analysis. The resulting correlation matrix was visualized in a heatmap format generated by the corrplot package of R [Corrplot: visualization of a correlation matrix; R package version 4.1.2. 2021].

## 3. Results

### 3.1. Growth and intestinal health

Prophylactic antimicrobials such us neomycin have been tranditionally used to prevent disease during the first month of life in dairy calves (Urie et al., 2018), with 40% of dairy operations in the US still relying on this as their main antimicrobial additive (USDA, 2018). However, in this study neomycin administration for either 14 days (ST) or 28 days (LT) had no effect in the growth and dry feed intake (*p* > 0.43; Supplementary Figures S1A,C), and only had a transient effect improving fecal consistency score on d 7 of life (*p* = 0.04; Supplementary Figure S1D).

### 3.2. Microbial diversity and relative abundance of bile-acid metabolizing bacteria

**Figure 1:**
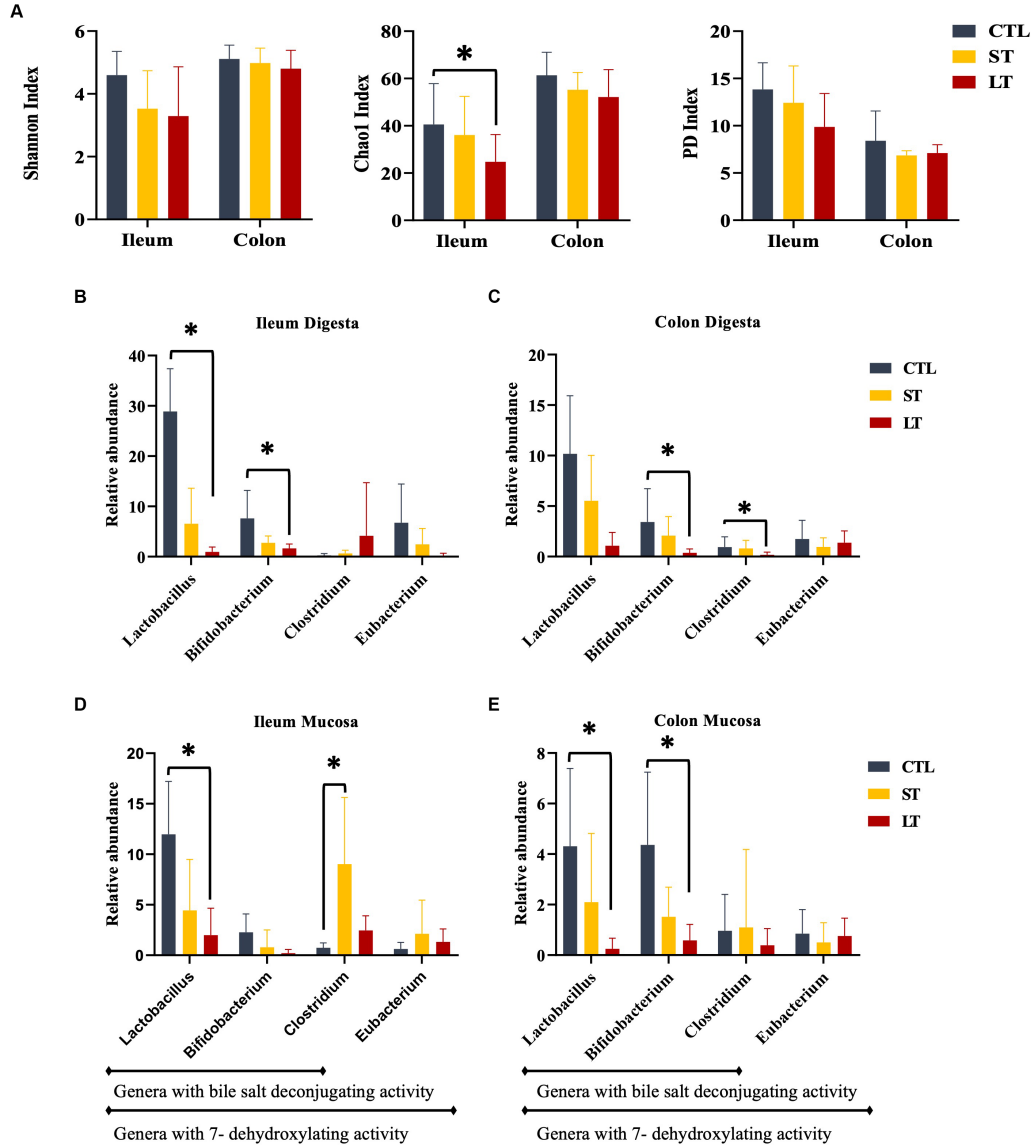
The effect of nonmedicated milk replacer (CTL), or prophylactic neomycin administration at 20 mg/kg per day for 14 days (Short-Term), or for 28 days (Long-Term) on microbial alpha diversity on ileum and colon **(A)**. Relative abundance of bile-acid metabolizing bacteria in ileum and colon digesta **(B, C)**, and tissue **(D, E)**. Treatment effects were tested with Kruskal Wallis and adjusted for multiple comparisons. Error bars denote SEM. *p < 0.05.

Calves that received prophylactic administration of neomycin for 28 days (LT) had reduced species richness (chao1 index) in the ileum tissue at the time of sampling on d 28 of the study (Figure 1B; *p* = 0.04) and tended to have reduced phylogenetic diversity (Figure 1B; *p* = 0.10). No differences were observed in the colon tissue (Figure 1B, *p* > 0.21). No differences between treatments were observed in the ileum digesta (Figure 1A, *p* > 0.32), and only a tendency was observed in colon digesta where CTL had higher phylogenetic diversity compared with ST (Figure 1A, *p* = 0.08).

Prophylactic administration of neomycin for either 14 or 28 days resulted in a shift in the composition of the intestinal microbiota in the ileum and colon of calves when compared with control (Supplementary Figure S2). The relative abundance of several bacterial genus groups known to possess genes for bile acid deconjugation and dihydroxylation. The relative abundance of *Lactobacillus*, and *Bifidobacterium* in ileum digesta, and *Lactobacillus*, *Bifidobacterium*, and *Clostridium* genus in colon digesta were decreased in LT compared with CTL (*p* < 0.05; Figure 1B). Additionally, the relative abundance of the *Lactobacillus* genus in Ileum tissue and *Lactobacillus* and *Bifidobacterium* genus in colon tissue were decreased in LT group compared with CTL (*p* < 0.04; Figure 1B). Lastly, in ileum tissue, the C*lostridium* genus was increased in ST compared with LT and CTL (*p* < 0.02; Figure 1B).

### 3.3. Bile acids profile in intestinal digesta and tissue

**Figure 2:**
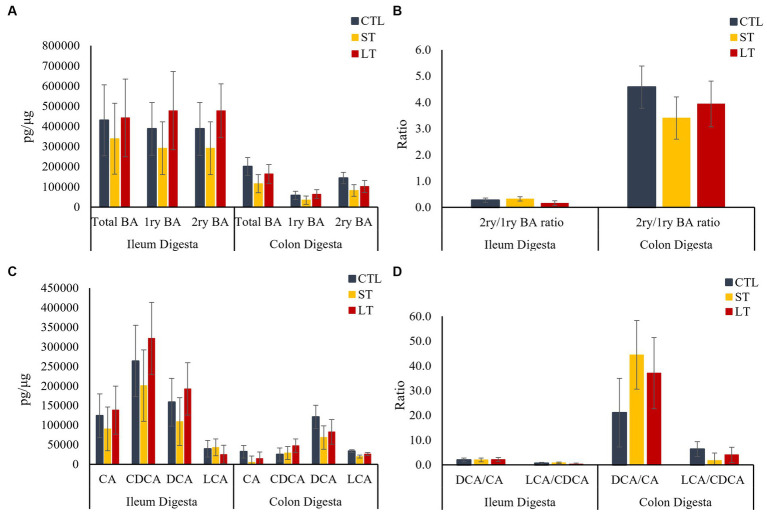
The effect of nonmedicated milk replacer (CTL), or prophylactic neomycin administration at 20 mg/kg per day for 14 days (Short-Term), or for 28 days (Long-Term) on concentrations (pg/μL) of **(A)** total bile acids (Total BA), primary bile acids (1ry BA), secondary bile acids (2ry BA); and **(C)** individual primary bile acids cholic acid (CA), chenodeoxycholic acid (CDCA), secondary bile acids deoxycholic acid (DCA) lithocholic acid (LCA) on ileum and colon digesta. In addition, Ratio of secondary to primary bile acids in ileum and colon digesta **(B)**, and ratio of DCA to CA, and LCA to CA **(D)**. Error bars denote SEM.

**Figure 3:**
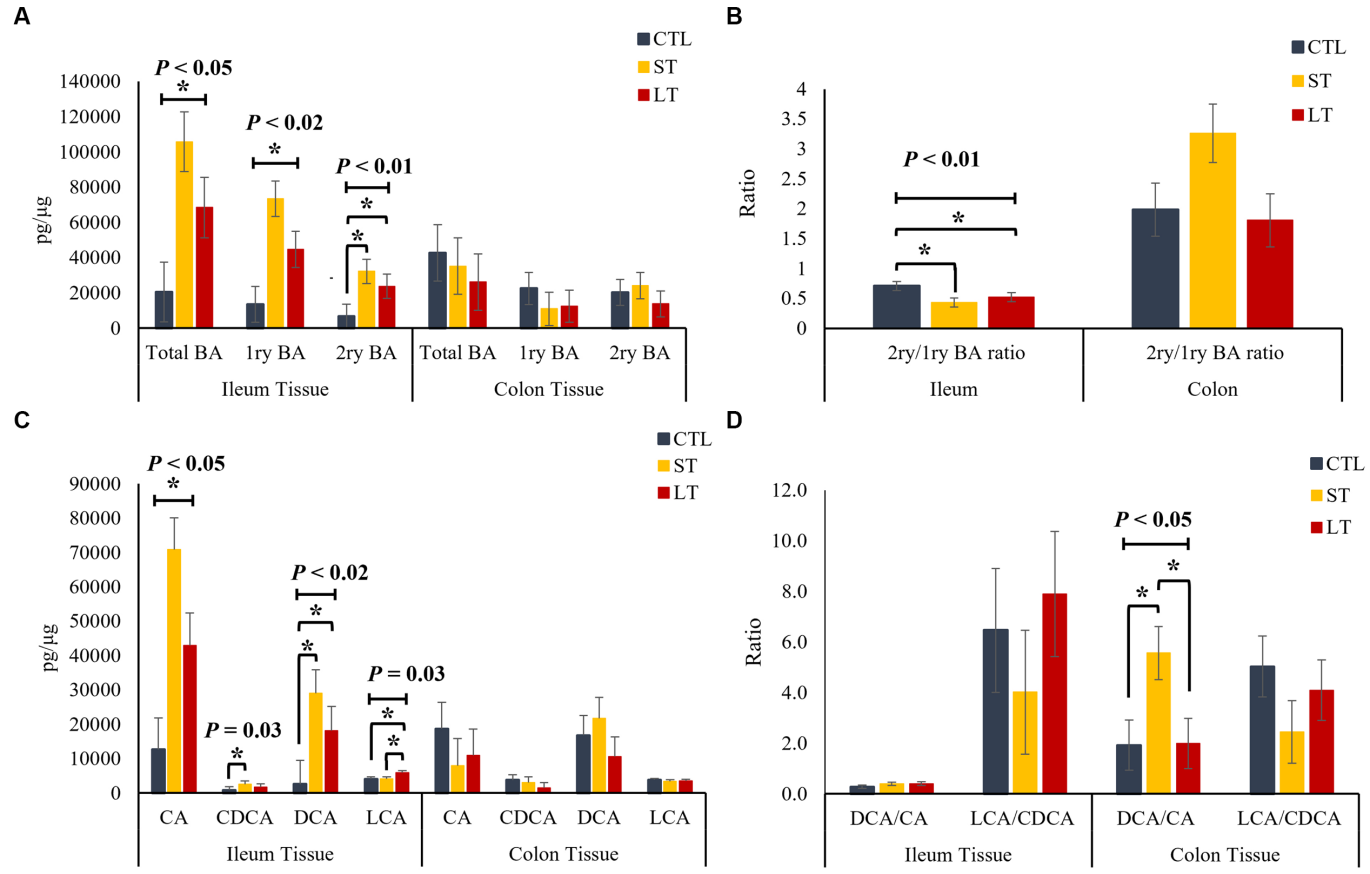
The effect of nonmedicated milk replacer (CTL), or prophylactic neomycin administration at 20 mg/kg per day for 14 days (Short-Term), or for 28 days (Long-Term) on concentrations (pg/μL) of **(A)** total bile acids (Total BA), primary bile acids (1ry BA), secondary bile acids (2ry BA); **(C)** individual primary bile acids cholic acid (CA), chenodeoxycholic acid (CDCA), secondary bile acids deoxycholic acid (DCA) lithocholic acid (LCA); and on the ratio of secondary to primary bile acids **(B)**, DCA to CA, and LCA to CA **(D)** in ileum and colon tissue. Error bars denote SEM.

To understand the impact of prophylactic administration neomycin on BA metabolism, the concentrations of BAs were measured in intestine, liver and plasma. All the individual primary and secondary BAs identified in the metabolomic analysis are depicted in Supplementary Figure S3. The different conjugated forms of a same BA were summed together and classified as total cholic acid (CA), chenodeoxycholic acid (CDCA), deoxycholic acid (DCA), or lithocholic acid (LCA) for all the compartments of the enterohepatic system and in plasma. No differences were observed in the concentration of primary, secondary, and total BA in ileum and colon digesta (*p* > 0.18; Figure 2A), however, a location effect was observed where the ileum had higher concentrations of total as well as primary BA compared to the colon (*p* < 0.03; Figure 2A). No treatment differences were observed in the concentrations of total primary BAs CA, CDCA, and secondary BAs DCA, and LCA (*p* > 0.36; Figure 2C). In addition, the ratio of secondary to primary BA as well as the ratio of DCA to CA in the colon was increased compared to the ileum (*p* < 0.01; Figures 2B,D). In ileum tissue, the concentration of primary, secondary, and total BA were increased in ST and LT compared with CTL (*p* < 0.05; Figure 3A). No treatment differences were observed for the BA profile in colon tissue (*p* > 0.62; Figure 3A). In addition, the concentrations of total primary BAs CA, CDCA, and secondary BAs DCA, and LCA were increased by ST compared with CTL (*p* < 0.05; Figure 3C) in ileum tissue but not in the colon. The ratio of secondary to primary BA was lower in ileum compared with colon (*p* < 0.01), and it was reduced in ST and LT compared with CTL in ileum tissue (*p* < 0.01; Figure 3B). Lastly, the ratio of DCA to CA was increased in ST compared with LT and CTL (*p* < 0.05; Figure 3D).

### 3.4. Bile acids profile in liver and plasma

**Figure 4:**
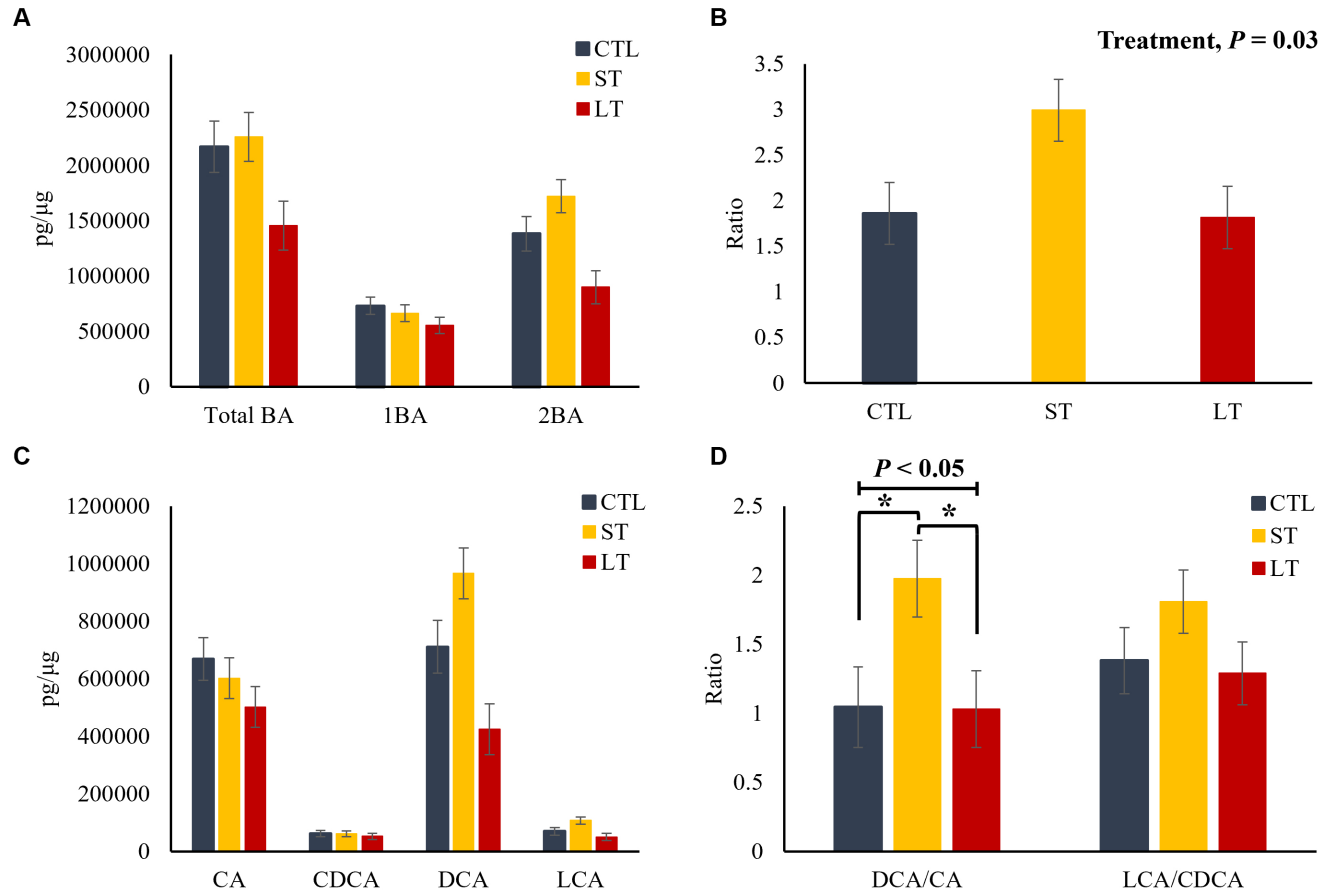
The effect of nonmedicated milk replacer (CTL), or prophylactic neomycin administration at 20 mg/kg per day for 14 days (Short-Term), or for 28 days (Long-Term) on concentrations (pg/μL) of **(A)** total bile acids (Total BA), primary bile acids (1ry BA), secondary bile acids (2ry BA); **(C)** individual primary bile acids cholic acid (CA), chenodeoxycholic acid (CDCA), secondary bile acids deoxycholic acid (DCA) lithocholic acid (LCA); and on the ratio of secondary to primary bile acids **(B)**, DCA to CA, and LCA to CA **(D)** in liver tissue. Error bars denote SEM.

**Figure 5:**
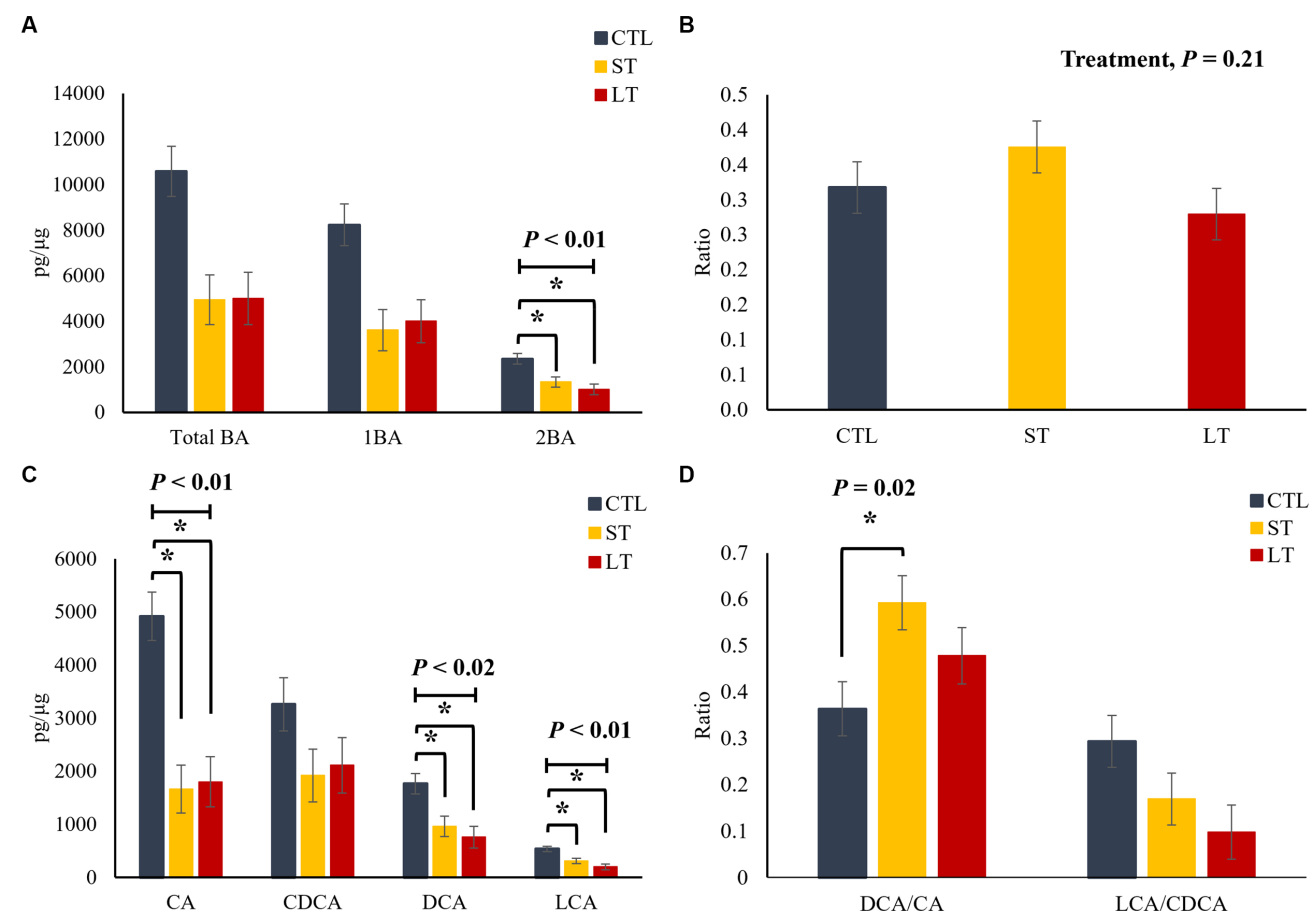
The effect of nonmedicated milk replacer (CTL), or prophylactic neomycin administration at 20 mg/kg per day for 14 days (Short-Term), or for 28 days (Long-Term) on concentrations (pg/μL) of **(A)** total bile acids (Total BA), primary bile acids (1ry BA), secondary bile acids (2ry BA); **(C)** individual primary bile acids cholic acid (CA), chenodeoxycholic acid (CDCA), secondary bile acids deoxycholic acid (DCA) lithocholic acid (LCA); and on the ratio of secondary to primary bile acids **(B)**, DCA to CA, and LCA to CA **(D)** in plasma. Error bars denote SEM.

In the liver, no differences among treatments were observed for total, primary and secondary BA (*p* > 0.24; Figure 4A), as well as for the individual primary and secondary BA (*p* > 0.22; Figure 4C). However, the ratio of secondary to primary BAs as well as the ratio of DCA to CA was increased in the ST group compared with CTL and LT (*p* < 0.04; Figures 4B,D). In plasma, ST and LT treatments had lower concentrations of secondary BA (*p* < 0.01; Figure 5A), CA, DCA, and LCA (*p* < 0.02; Figure 5C) compared with CTL. No differences were observed for the ratio of total secondary to primary BA (*p* = 0.21; Figure 5B), however, there was a treatment effect on the ratio of DCA to CA in plasma was higher in ST compared with CTL (*p* = 0.02; Figure 5D).

### 3.5. Expression of BA associated genes

**Figure 6:**
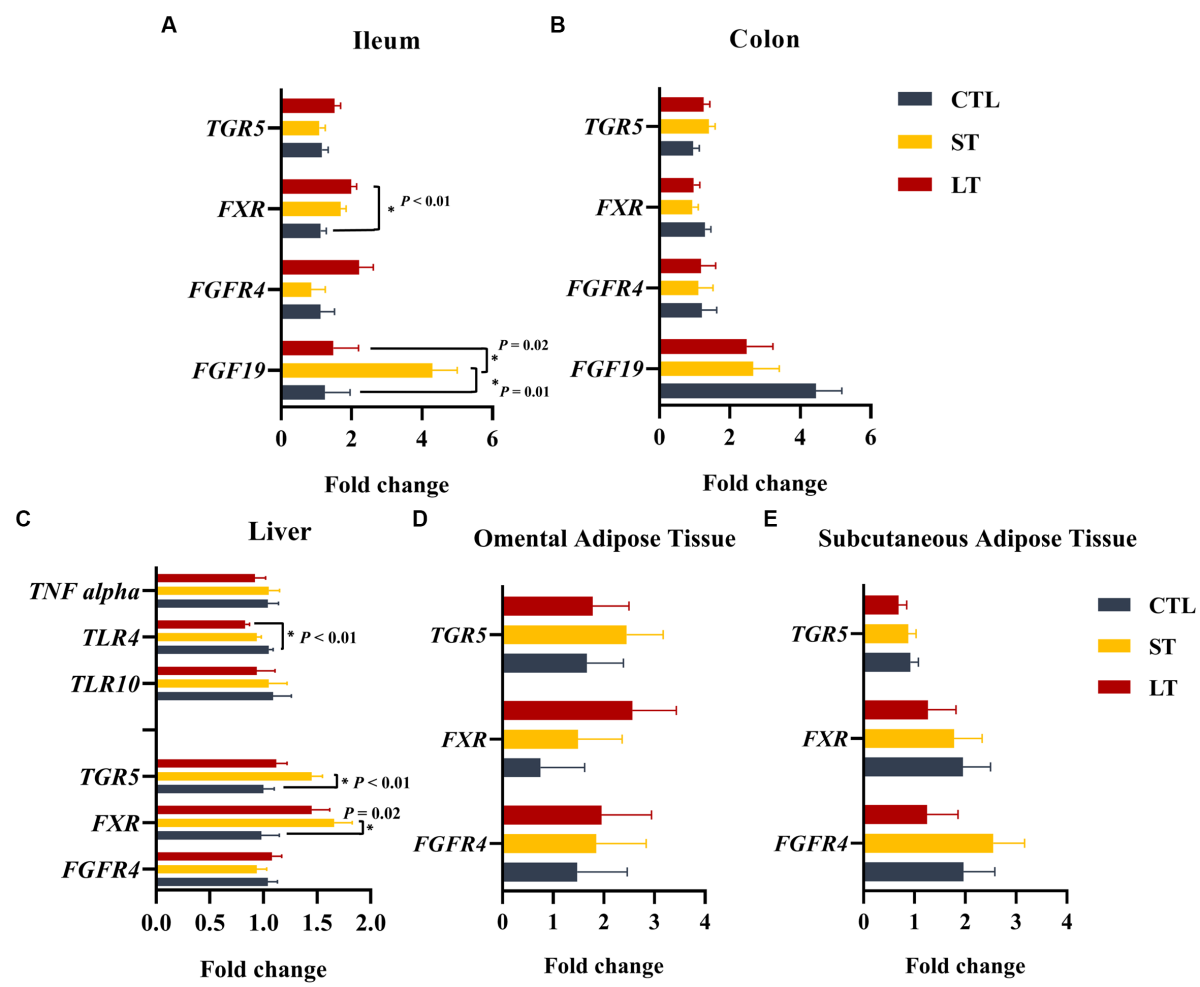
The effect of nonmedicated milk replacer (CTL), or prophylactic neomycin administration at 20 mg/kg per day for 14 days (Short-Term), or for 28 days (Long-Term) on transcript abundance of genes related to bile acid metabolism on ileum **(A)**, colon **(B)**, liver tissue **(C)**, omental adipose tissue **(D)**, and peripheral adipose tissue **(E)**. Error bars denote SEM.

The uptake and transport of BAs into portal circulation is tightly regulated by a group of BA dedicated receptors, the nuclear receptor farnesoid × receptor (*FXR*), and the g protein-coupled receptor (*TGR5*). In addition, fibroblast growth factor 19 (*FGF19*) is a growth factor produced by intestinal entero-endocrine cells, and its production is mediated by *FXR.* Additionally, fibroblast growth factor receptor 4 is the dedicated receptor for *FGF19*. The genes *FXR*, *TGR5*, *FGF19*, and *FGFR4* were constitutively expressed in the ileum and colon. The transcript abundance of *FXR* was higher in ileum compared with the colon (*p* < 0.01; Figures 6A,B). Furthermore, in the ileum, the transcript abundance of *FXR* was higher for the LT group compared with CTL (*p* < 0.04; Figure 6A), and *FGF19* was higher for ST (*p* < 0.04; Figure 6A).

In the liver, BA synthesis and reuptake from portal circulation is controlled by *FXR* and *TGR5,* respectively. The expression of *FXR* and *TGR5* in the liver was increased in the ST group compared with CTL (*p* < 0.02; Figure 6C). In addition, the expression of *TLR4*, which is known to be downregulated by *FXR* in the liver (Renga et al., 2009), was reduced in LT but not ST compared with CTL (*p* < 0.01; Figure 6C). Lastly, adipose tissue also constitutively expressed FXR, TGR5, and FGFR4 in both subcutaneous and omental sites. The transcript abundance of *FGFR4* was higher in omental adipose tissue compared to subcutaneous (*p* = 0.05), and *TGR5* was higher in subcutaneous adipose tissue compared to omental (*p* = 0.01), however, no treatment differences were observed (Figures 6D,E).

### 3.6. Transcript abundance of genes associated with lipid metabolism and immune function in adipose tissue

**Figure 7:**
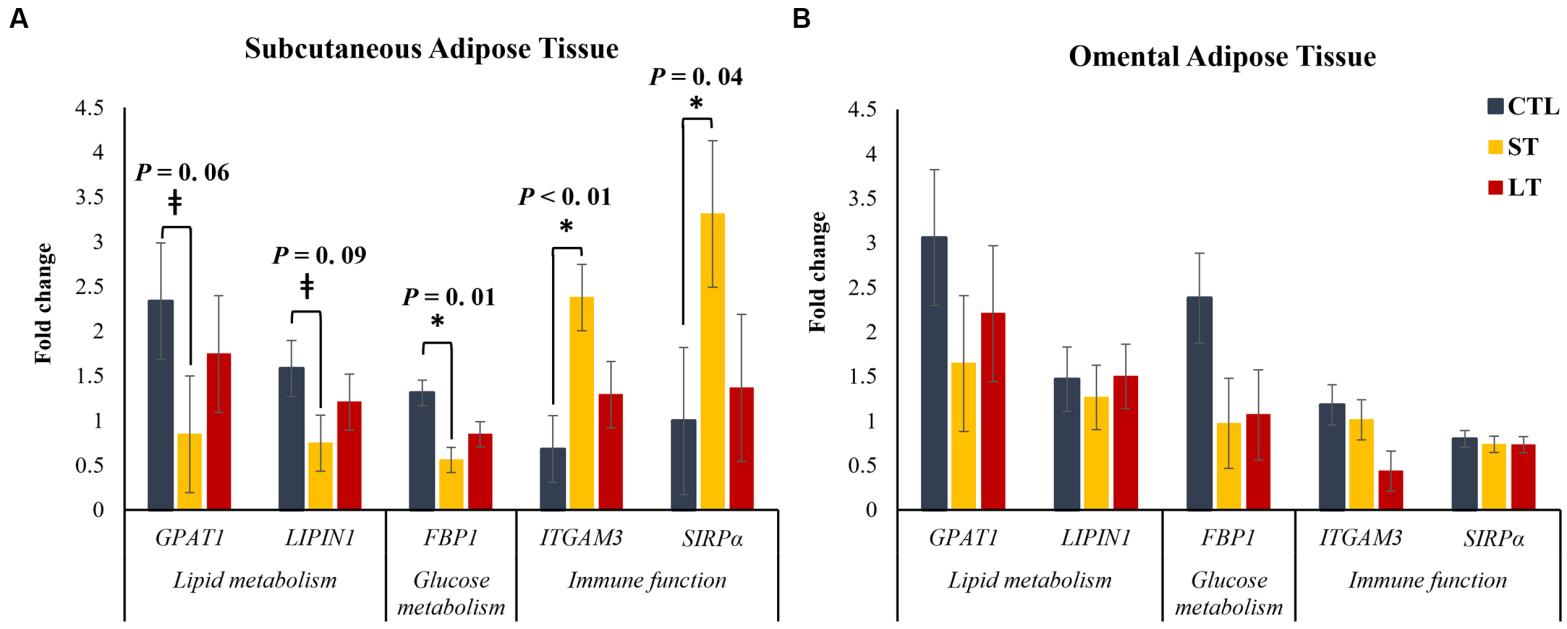
The effect of nonmedicated milk replacer (CTL), or prophylactic neomycin administration at 20 mg/kg per day for 14 days (Short-Term), or for 28 days (Long-Term) on transcript abundance of genes related to lipid metabolism and immune function in **(A)** Subcutaneous or **(B)** Omental adipose tissue differentially expressed between treatments. Error bars denote SEM.

From a transcriptional study of 48 genes involved in lipid metabolism, glucose metabolism, and immune activation, five genes were differentially expressed between treatments, and are presented in Figure 7. There was a tendency in peripheral adipose tissue, but not in omental adipose tissue for the genes glycerol-3-phosphate acyltransferase (*GPAT1*) and *LPIN,1,* two genes that encode for triacylglycerol biosynthesis enzymes, to be decreased in ST compared with CTL (*p* < 0.09; Figure 7A). In addition, the transcript abundance of the gene *FBP1,* which encodes for the gluconeogenic rate-limiting enzyme fructose-1,6-bisphosphatase, was decreased in ST compared to CTL (*p* = 0.01; Figure 7A) in peripheral adipose tissue, but not in omental adipose tissue. There was a treatment by tissue interaction for the transcript abundance of the gene *PGK1* that encodes for phosphoglycerate kinase 1, a critical enzyme in the process of glycolysis (*p* = 0.05; Supplementary Table S2), with ST having increased transcript abundance compared with CTL. Lastly, the transcript abundance of the genes *ITGAM3* and *SIRPα*, two genes related to immune activation in macrophages, were increased in ST compared with CTL (*p* < 0.04; Figure 7A) in peripheral adipose tissue.

### 3.7. Correlations between microbiota and bile acids in digesta and mucosa

**Figure 8:**
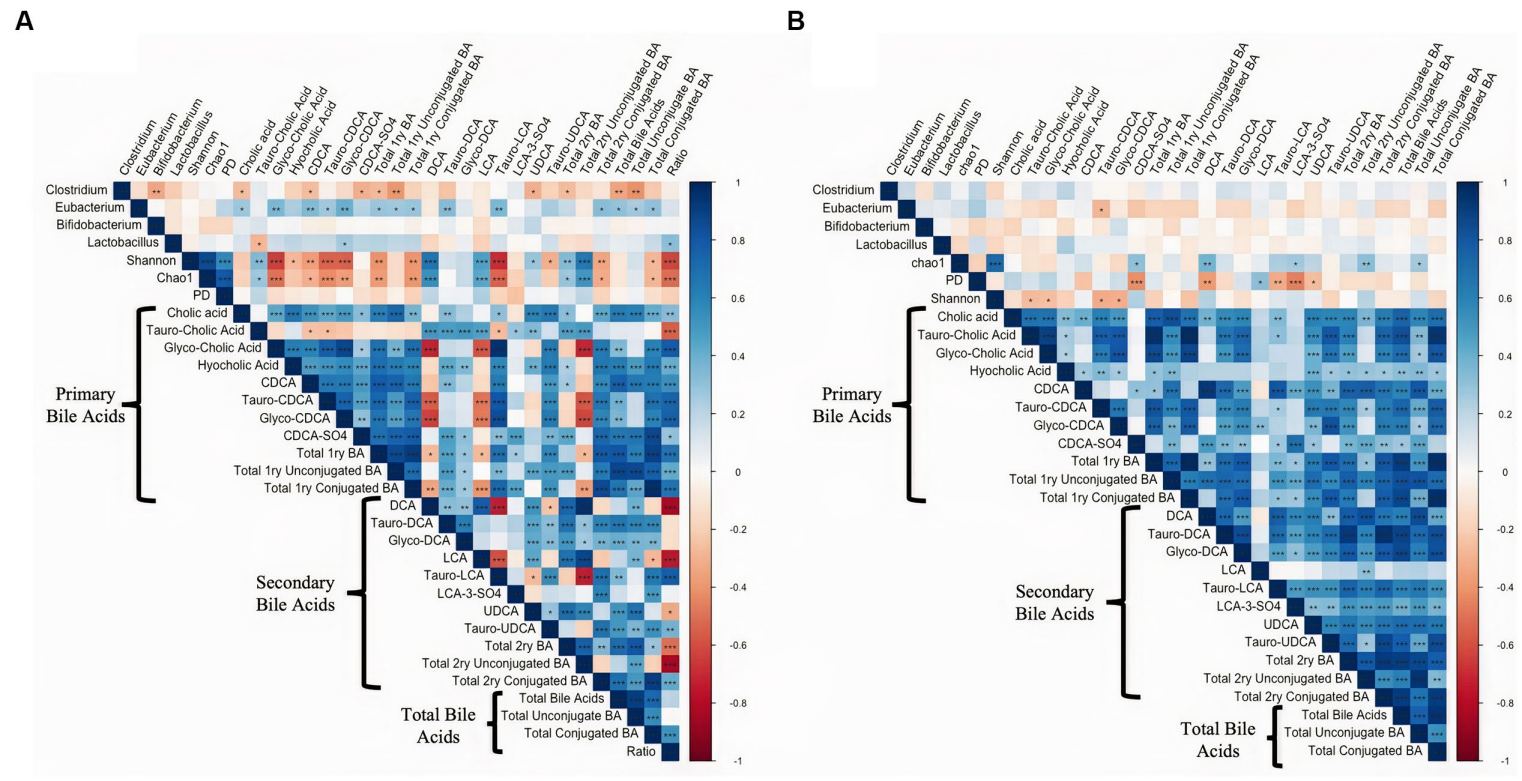
Spearman rank correlation analysis comparing alpha diversity measures and the bacterial relative abundance with all the individual primary bile acids, secondary bile acids, and their respective unconjugated and conjugated forms in **(A)** intestinal digesta and **(B)** intestinal tissue. Data is graphically presented in a square plot heatmap. Correlations are indicated by a color scale denoting whether the correlation is positive (closer to 1, blue squares) or negative (closer to −1, red squares). Statistically significant correlations are indicated by a *. *p ≤ 0.05, **p ≤ 0.01, ***p ≤ 0.001.

Spearman rank correlation analysis was conducted comparing alpha diversity measures and the bacterial relative abundance presented in Figure 1 with all the individual primary bile acids, secondary bile acids, and their respective unconjugated and conjugated forms in intestinal tissue, and digesta are presented in Figure 8. In the digesta, *Eubacterium* was positively correlated with several primary (*p* ≤ 0.04, Spearman’ ρ ≥ 0.34) and the secondary Bas Tauro-DCA, and Tauro-LCA (*p* ≤ 0.01, Spearman’ ρ ≥ 0.32). Additionally, members of the *Clostridium* genus were negatively correlated with the total unconjugated primary BAs (*p* ≤ 0.01, Spearman’ ρ ≥ 0.39) as well as total unconjugated BA (*p* ≤ 0.01, Spearman’ ρ ≥ 0.40). The Shannon index of microbial diversity, as well as the Chao1 index of species richness were positively correlated with the concentrations of secondary BAs DCA (*p* ≤ 0.01, Spearman’ ρ ≥ 0.54), and LCA (*p* ≤ 0.01, Spearman’ ρ ≥ 0.46), and total 2ry unconjugated BAs (*p* ≤ 0.01, Spearman’ ρ ≥ 0.51). Additionally, Shannon and Chao1 indexes were negatively correlated with the concentrations of Tauro conjugated LCA (*p* ≤ 0.01, Spearman’ ρ ≥ 0.53). In the intestinal tissue samples species richness was positively correlated with concentrations of the secondary BA DCA (*p* < 0.01, Spearman’ ρ = 0.37), and with total unconjugated secondary BAs (*p* = 0.01, Spearman’ ρ = 0.32). Lastly, phylogenetic diversity index was negatively correlated with the sulfated form of CDCA (*p* ≤ 0.01, Spearman’ ρ = 0.42), and with the secondary BAs DCA, LCA, and UDCA (*p* ≤ 0.01, Spearman’ ρ ≥ 0.31).

## 4. Discussion

Prophylactic antimicrobials have been traditionally used to prevent enteric infections in calves and to improve growth rates during the preweaning period. In recent years, this practice has been challenged given its demonstrated low efficacy in preventing disease, as well as concerns regarding antimicrobial resistance (Windeyer et al., 2014; Aghakeshmiri et al., 2017). In this study, we demonstrate that the prophylactic use of the antimicrobial neomycin is implicated in the dysregulation of BA metabolism by altering its profile and tissue distribution, resulting in changes in transcript abundance of genes related to metabolic function and inflammation in peripheral adipose tissue and liver.

The prophylactic administration of neomycin for either 14 or 28 days resulted in a minor, yet significant reduction in species richness in the ileum, with no significant changes in the colon. This intervention also resulted in a reduction in the relative abundance of the Firmicutes phylum that was replaced by an increase in Proteobacteria. Similarly, several studies have shown that although sub-therapeutic doses of prophylactic antimicrobials result in less consistent shifts in microbial composition, it induces marked alterations in lipid and BA metabolism (De Aguiar Vallim et al., 2013; Ipharraguerre et al., 2018; Cai et al., 2022). This effect may be carried out by specific alterations to the bacterial composition, leading to changes in the production and profile of short-chain fatty acids, increased intestinal permeability, and systemic inflammation. Additionally, it is linked with changes in the abundance of several bacterial groups reported to express genes for deconjugation (bile salt hydrolases; BSH) and dehydroxylation of primary BA (BA-inducible genes; BAI). These include members from the genus *Clostridium, Enterococcus, Bifidobacterium, and Lactobacillus* (Patel et al., 2010; Yao et al., 2018; Cai et al., 2022). Although a consistent microbial enterotype has not been established across published literature, suppression of bacteria with the capacity to modify primary BA leads to changes in the luminal concentration and reuptake of BA, lipid absorption, and BA signaling. In this study, we observed that neomycin administration resulted in a marked reduction in the abundance of bacterial groups known to contain BSH and BAI genes, particularly of *Lactobacillus* and *Bifidobacterium.* This is in line with the findings from Ipharraguerre et al. (2018), where subtherapeutic administration of antimicrobials to weaned piglets resulted in a small reduction in microbial diversity and a reduction in the abundance of bacteria from the *Lactobacillus* genus. The short-term treatment (ST) had a limited impact on these bacterial communities, suggesting that after interruption of neomycin administration, their abundance started to recover, although 14 days was not enough to restore pre-antibiotic conditions. Further research is required to determine if prophylactic neomycin administration does in fact leads to a loss of function in bile acid microbial transformation by directly assessing expression of BSH and BAI genes.

We then investigated the impact of neomycin administration on BA synthesis and circulation in enterohepatic and systemic circulation. The terminal ileum is the main site of active reuptake of BA in the intestine, where approximately 95% are recirculated back *via* the portal vein to the liver. In our study, while no changes were observed in the luminal concentrations of BA, there was an increase in their uptake in the ileum of both ST and LT groups compared with CTL, suggesting that the neomycin-induced changes in the abundance of BA metabolizing bacteria upregulated the reuptake of BA by the ileum mucosa. Additionally, based on the correlation analysis, it appears that lower microbial diversity in the digesta was correlated with a shift in the profile of BAs resulting in a reduction in secondary BAs with a concomitant increase in primary BAs. Furthermore, the reduction in the abundance of BSH and BAI-containing bacteria might have increased the availability of conjugated BA, which are preferentially transported across the gut by BA transporters, partly explaining the increase in ileum reuptake. This is also supported by the results from the correlation analysis that suggests that a reduction in microbial diversity in the digesta was positively correlated with an increase in Taurine conjugated BAs and negatively correlated with the concentrations of unconjugated secondary BAs. This suggests that the neomycin-driven changes in microbial diversity and composition resulted in changes in the BA profile ultimately affecting their intestinal reuptake. Furthermore, the increased reuptake of BAs upregulated the expression of the nuclear receptor FXR in calves that were still receiving neomycin at the time of dissection. Once BAs are absorbed across the gut, the majority are transported back to the liver for conversion back to their primary form and conjugated with taurine or glycine (Falany et al., 1994). In the liver, BA interact with the nuclear receptor FXR to downregulate their synthesis in a negative feedback system that suppresses the expression of the cytochrome P450 enzyme 7α-hydroxylase preventing the first step in the conversion of cholesterol into primary BA (Lefebvre et al., 2009). The agonistic potency of BA on their cognate receptors FXR and TGR5 is higher for DCA than for CA, which in turn affects activation and downstream signaling cascades (Chávez-Talavera et al., 2017). In our study, although we did not observe changes in the concentration of primary or secondary BA in the liver, there was an increase in the ratio of secondary to primary BA in the ST group due to an increase in DCA, and reduction in CA, which might be responsible for the increased expression of both FXR and TGR5. Taken together, this data suggest that the changes in the abundance of BSH and BAI-containing bacteria caused by neomycin administration altered the distribution of the BA pool within the enterohepatic system by increasing ileal reuptake, and changing the expression of genes responsible in BA synthesis, and transport in the liver.

Several studies in a variety of animal models have shown a direct link between antimicrobial exposure in early life with a concomitant increase in adiposity and aberrant metabolic function in adulthood (Vallianou et al., 2021), with dysregulation of BA metabolism serving as one of the proposed mechanisms. As previously mentioned, BA serve as metabolic integrators, conveying information from the intestinal milieu to peripheral tissues. Therapeutic doses of antimicrobials have been shown to alter BA profile and tissue distribution, as well as the expression of downstream genes involved in energy metabolism and immune function in rodents (Swann et al., 2011; Cai et al., 2022) and piglets (Ipharraguerre et al., 2018). A small proportion of BAs escape the enterohepatic system and enter systemic circulation, where they interact with TGR5 and FXR expressed in adipose tissue and skeletal muscle tissue to activate transcriptional networks and signaling cascades controlling the expression and activity of genes involved in lipogenesis, gluconeogenesis, and inflammation (Lefebvre et al., 2009; Li et al., 2013; Ipharraguerre et al., 2018). In our study, while prophylactic administration of neomycin increased ileal reaptake of BA, it reduced the concentration of primary (CA) and secondary BA (DCA and LCA) in the plasma of preweaned calves on d 28 of life. Additionally, although no statistically significant differences were observed in the expression of FXR and TGR5 in adipose tissue, administration of neomycin for 14 days (ST), but not 28 days (LT), was linked to changes in the transcript abundance of genes related to phospholipid synthesis, lipogenesis, gluconeogenesis, and immune activation in peripheral adipose tissue. However, only five out of the 48 genes tested in this study were significantly different between treatments. Thus, to establish a causational link between the dysregulation of BA metabolism as a result of neomycin administration and alterations in adipose lipid metabolism and systemic inflammation, further research is required.

Lastly, this study revealed that although calves in the ST group were not receiving neomycin at the time of slaughter (day 28), they still exhibited alterations in BA concentrations along with changes in the expression of genes involved in BA metabolism in ileum, liver, and peripheral tissues. Furthermore, despite our initial hypothesis, the extent of these changes observed for ST were greater than in the LT group, particularly with regards to ileal reuptake of BA, expression of BA genes in the liver, and gene expression in peripheral adipose tissues. These findings imply that antimicrobial dosing led to changes in BA and metabolic function that persisted even after neomycin administration was discontinued, and that the distinct intestinal microbiota in the ST group could have induced an expansion of BA signaling. Typically, prophylactic antimicrobial therapy leads to a decrease in microbial diversity. However, upon cessation of antimicrobial administration, a new wave of microbial colonization occurs, and the course of recolonization during the initial post-antimicrobial period can significantly impact the long-term microbiome structure in and health outcomes of the host (Schwartz et al., 2020). In our study, the microbial diversity and relative abundance of bacteria with BSH and BAI suggest that interruption of neomycin administration resulted in a reorganization of these bacterial populations, which might have resulted in changes in the BA profile and signaling.

In summary, the findings of this study demonstrate that prophylactic administration of the antimicrobial neomycin resulted in changes in BA metabolism by altering its profile and tissue distribution. Additionally, we propose a possible link between dysregulated BA profile and tissue distribution, with changes in the transcript abundance of genes related to lipid metabolism, immune activation, and inflammation in adipose tissue and liver. Importantly, these alterations in BA metabolism and gene expression are still observed even after ceasing neomycin administration. Future studies should expand on these results to determine if the observed changes in transcript abundance correlate with physiological changes in adipose tissue function. Lastly, future studies should examine the enduring effects of antimicrobial derived alterations of the intestinal microbiota during early life to understand if certain metabolic and inflammatory disorders in adult cows stem from maladaptations during early development.

## Data availability statement

The data presented in the study are deposited in the NCBI SRA repository, accession number PRJNA1007748.

## Ethics statement

The animal study was approved by Canadian Council of Animal Care. The study was conducted in accordance with the local legislation and institutional requirements.

## Author contributions

All authors listed have made a substantial, direct, and intellectual contribution to the work and approved it for publication.

## Conflict of interest

The authors declare that the research was conducted in the absence of any commercial or financial relationships that could be construed as a potential conflict of interest.

## Publisher’s note

All claims expressed in this article are solely those of the authors and do not necessarily represent those of their affiliated organizations, or those of the publisher, the editors and the reviewers. Any product that may be evaluated in this article, or claim that may be made by its manufacturer, is not guaranteed or endorsed by the publisher.
