# Supplementary material for: Prophylactic feeding of neomycin to Holstein calves alters gut microbiota, bile acid metabolism, and expression of genes involved in immunometabolic regulation
Source: Front Microbiol. 2023 Aug 31;14:1210142. doi: 10.3389/fmicb.2023.1210142 (PMC10500837; doi:10.3389/fmicb.2023.1210142)
Supplement: Supplementary file 1 [file Data_Sheet_1.docx]

**Supplementary Table 1: List of primers for bile acid genes.**

| **Gene** | **Forward Primer** | **Reverser Primer** | **Amplicon Size** |
| --- | --- | --- | --- |
| *FXR* | CCCAGGGCTTTGAAAGTTCACC | TCCAAGAACCCTAGAGATGCGG | 122 |
| *TGR5* | GTCAGGACACCAGACGTCGC | GCAGCTAGCAACCGGCCTTT | 137 |
| *FGF19* | ACGGCAAGATGCAAGGACTG | ACACGTTGTAGCCGTCAGG | 84 |
| *FGFR4* | ACACCTGCCTCGTGGAGAAT | TGCAGAGCAGTTCCACGTCA | 150 |

**Supplementary Table 2:** The effect of nonmedicated milk replacer (CTL), or prophylactic neomycin administration at 20 mg/kg per day for 14 days (Short-Term), or for 28 days (Long-Term) on transcript abundance of genes related to lipid metabolism, carbohydrate metabolism, and immune function on adipose tissue differentially expressed between treatments.

|  |  | **Trt** | | |  | **Tissue** | |  |  |  | ***P*-value** | | |
| --- | --- | --- | --- | --- | --- | --- | --- | --- | --- | --- | --- | --- | --- |
| **Function** | **Gene** | **CTL** | **ST** | **LT** |  | **OME** | **SCT** |  | **SEM** |  | **Treatment** | **Tissue** | **Treatment × Tissue** |
| Lipid metabolism | *GPAT1* | 2.59 | 1.11 | 1.97 |  | 2.2 | 1.58 |  | 0.586 |  | 0.098 | 0.159 | 0.7753 |
|  | *LIPIN1* | 1.59 | 0.98 | 1.37 |  | 1.38 | 1.25 |  | 0.142 |  | 0.071 | 0.519 | 0.7092 |
| Glycolisis | *PGK1* | 0.94 | 1.23 | 0.88 |  | 0.8 | 1.24 |  | 0.172 |  | 0.348 | <0.01 | 0.054 |
| Immunity | *ITGAM3* | 0.93 | 1.66 | 0.86 |  | 0.85 | 1.45 |  | 0.195 |  | 0.064 | 0.05 | 0.045 |
|  | *SIRPα* | 0.91 | 1.93 | 1.05 |  | 0.76 | 1.84 |  | 0.425 |  | 0.076 | <0.01 | 0.0584 |


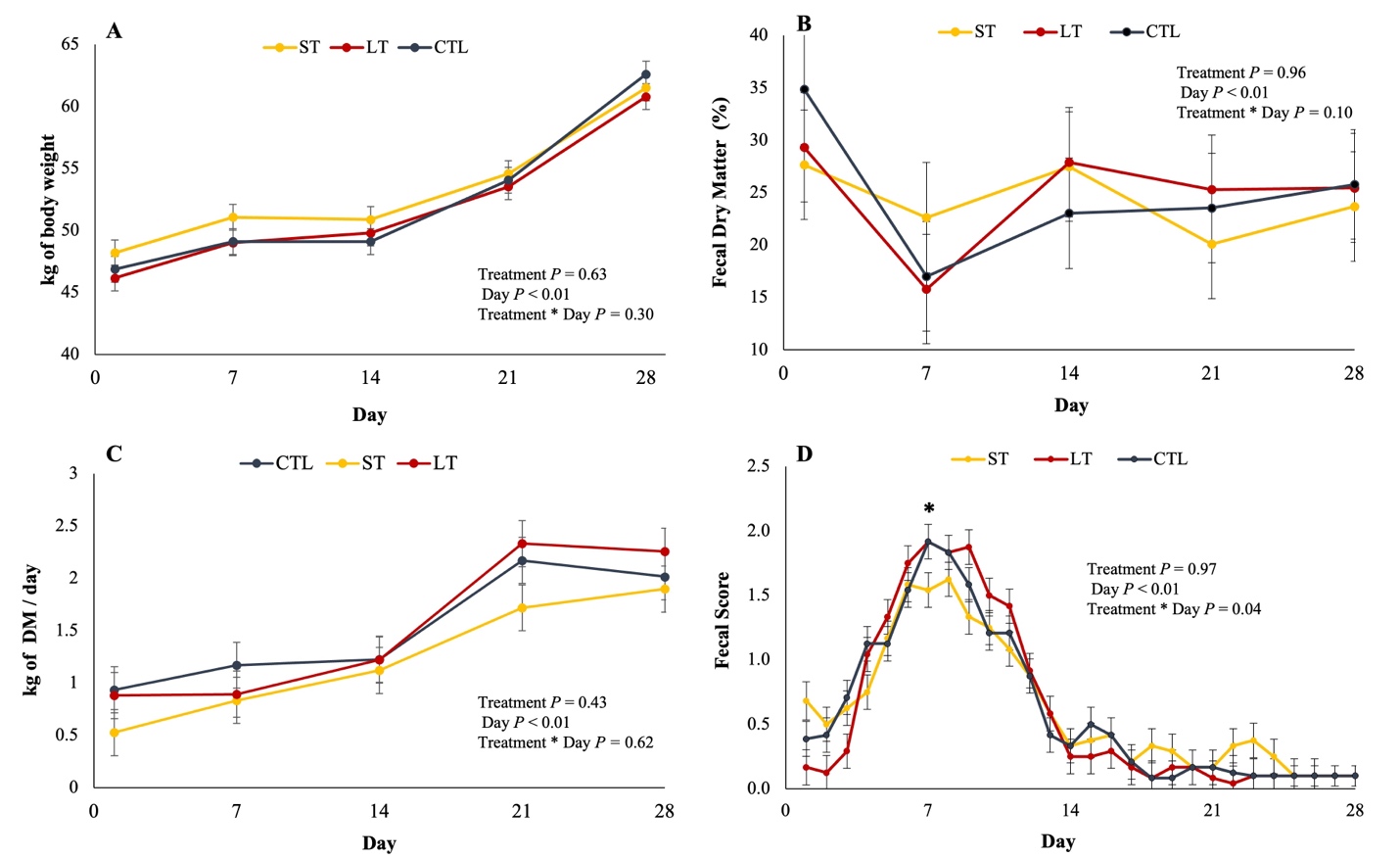


**Supplementary Figure 1:** The effect of nonmedicated milk replacer (CTL), or prophylactic neomycin administration at 20 mg/kg per day for 14 days (Short-Term), or for 28 days (Long-Term) on kg of body weight gain, kg of dry matter (DM) intake of starter feed, fecal scores, and fecal DM per day.


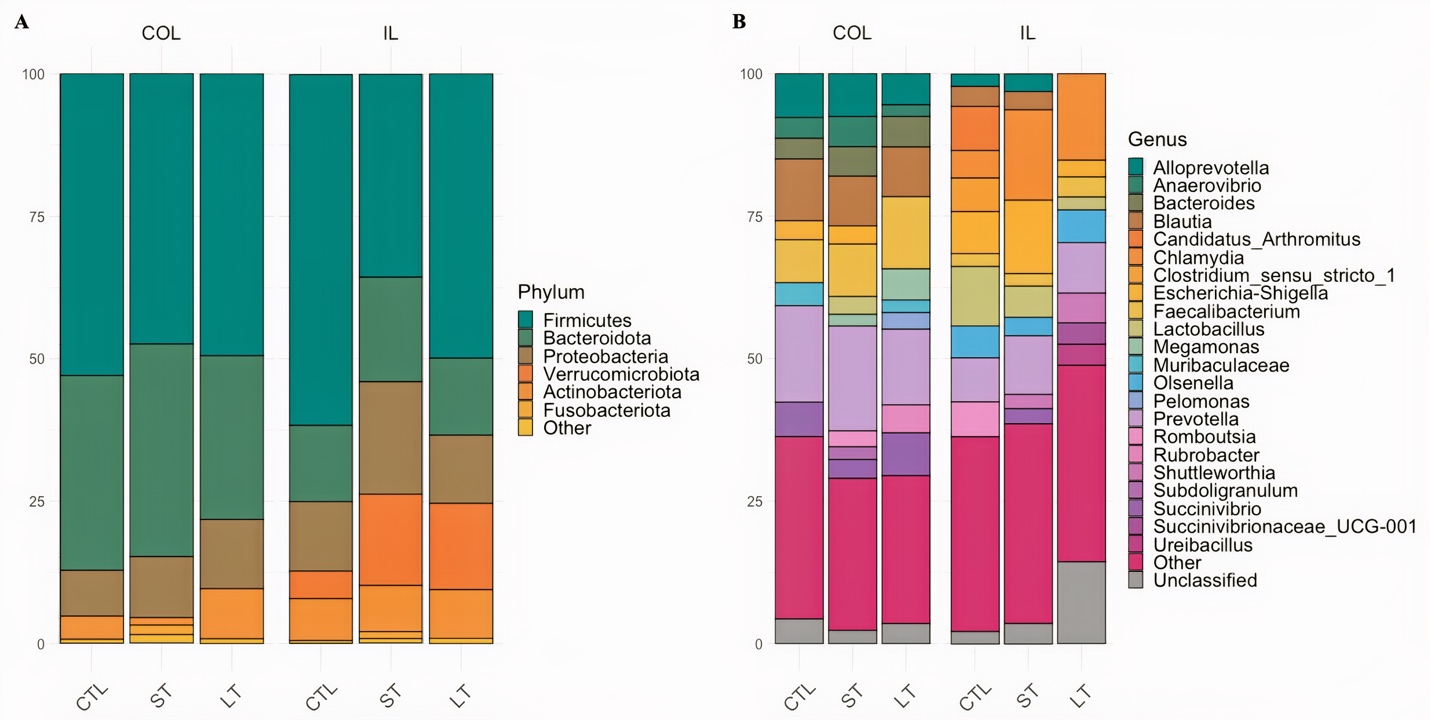


**Supplementary Figure 2:** The effect of nonmedicated milk replacer (CTL), or prophylactic neomycin administration at 20 mg/kg per day for 14 days (Short-Term), or for 28 days (Long-Term) on relative abundance of bacteria (%) in colon and ileum at the phylum (A) and genus (B) level.


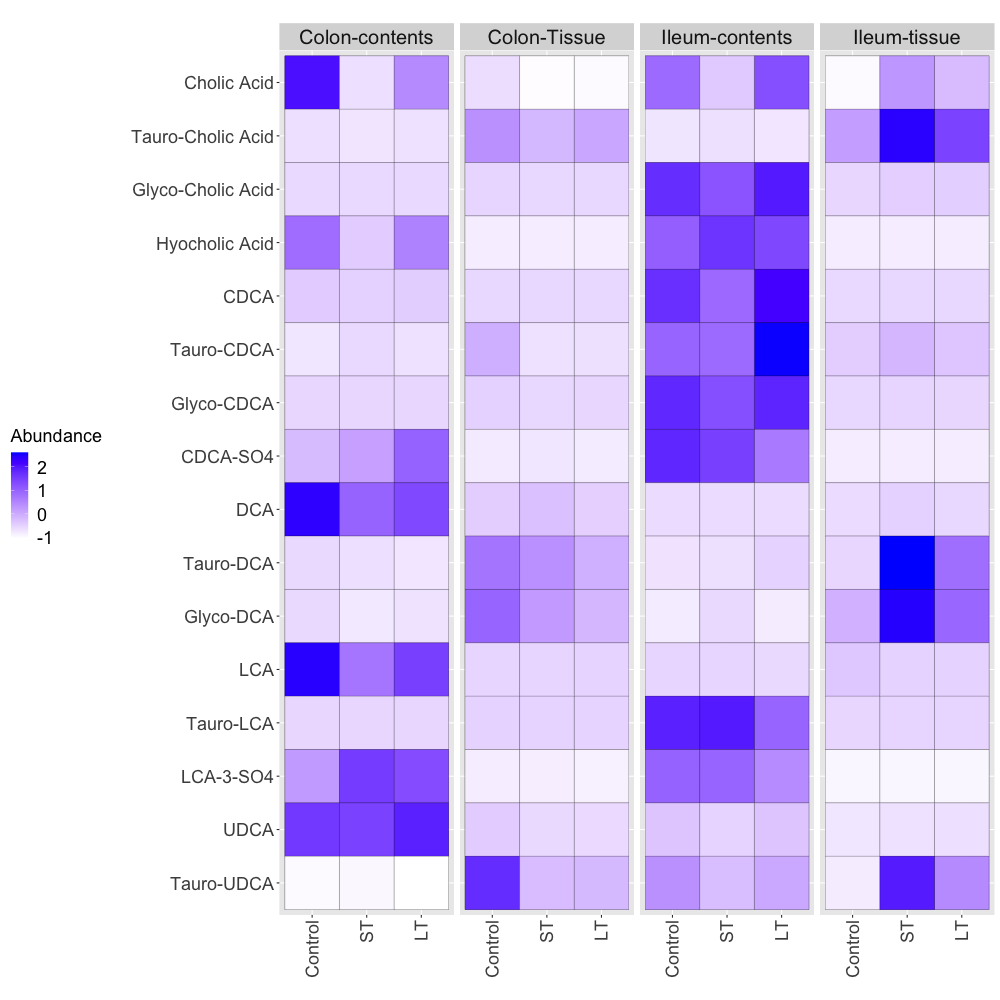


**Supplementary Figure 3:** The effect of nonmedicated milk replacer (CTL), or prophylactic neomycin administration at 20 mg/kg per day for 14 days (Short-Term), or for 28 days (Long-Term) on individual primary and secondary bile acids (BA) in ileum and colon from content and tissue samples. The concentration of each individual BA was scaled and plotted as heatmap, and colors represent the changes in abundance of each BA relative to the mean.
